# Variations in hospital standardised mortality ratios (HSMR) as a result of frequent readmissions

**DOI:** 10.1186/1472-6963-12-91

**Published:** 2012-04-04

**Authors:** Wim F van den Bosch, Peter Spreeuwenberg, Cordula Wagner

**Affiliations:** 1 St. Antonius Hospital, P.O. Box 2500, 3430, EM Nieuwegein, the Netherlands; 2 NIVEL, the Netherlands Institute for Health Services Research, P.O. Box 1568, Utrecht, the Netherlands; 3 VU University Medical Centre, De Boelelaan 1117, 1081, HV Amsterdam, the Netherlands

## Abstract

**Background:**

We investigated the impact that variations in the frequency of readmissions had upon a hospital's standardised mortality ratio (HSMR). An adapted HSMR model was used in the study. Our calculations were based on the admissions of 70 hospitals in the Netherlands during the years 2005 to 2009.

**Methods:**

Through a retrospective analysis of routinely collected hospital data, we calculated standardised in-hospital mortality ratios both by hospital and by diagnostic group (H/SMRs) using two different models. The first was the Dutch 2010 model while the second was the same model but with an additional adjustment for the readmission frequency. We compared H/SMR outcomes and the corresponding quality metrics in order to test discrimination (c-statistics), calibration (Hosmer-Lemeshow) and explanatory power (pseudo-R^2 ^statistic) for both models.

**Results:**

The SMR outcomes for model 2 compared to model 1, varied between -39% and +110%. On the HSMR level these variations ranged from -12% to +11%. There was a substantial disagreement between the models with respect to significant death on the SMR level as well as the HSMR level (~ 20%). All quality metrics comparing both models were in favour of model 2. The susceptibility to adjustment for readmission increased for longer review periods.

**Conclusions:**

The 2010 HSMR model for the Netherlands was sensitive to adjustment for the frequency of readmissions. A model without this adjustment, as opposed to a model with the adjustment, produced substantially different HSMR outcomes. The uncertainty introduced by these differences exceeded the uncertainty indicated by the 95% confidence intervals. Therefore an adjustment for the frequency of readmissions should be considered in the Netherlands, since such a model showed more favourable quality metric characteristics compared to a model without such an adjustment. Other countries could well benefit from a similar adjustment to their models. A review period of the data collected over the last three years, at least, is advisable.

## Background

Hospital standardised mortality ratios (HSMRs) are widely used as an indicator to assess and improve the quality of care. HSMRs have been calculated in the UK, USA, Canada, Australia and the Netherlands [1]. HSMR outcomes fluctuate around 100. For example in the UK the outcomes range from 72 to 118 and in the Netherlands from 62 to 142 [1,2]. The Dutch HSMR model was developed by the Dr. Foster Intelligence Unit in London in co-operation with Kiwa Prismant in the Netherlands. The model adjusts for patient casemix factors including age, sex and diagnostic group. It is based upon the national medical registration data (LMR). In October 2010 the 'Dutch Hospital Data' group (DHD) distributed the hospital specific, 'H/SMR report 2007-2009 with detailed information on diagnostic groups and patient categories', among Dutch hospitals. In this way, each hospital obtained an insight into their own standardised mortality ratios (SMRs) for 50 diagnostic groups and had the opportunity to work on improving patient safety.

HSMRs are also made publicly available in countries, for example in the UK's '2010 Dr Foster Hospital Guide'[2]. However questions have been raised about how reliable, valid and applicable the model really is [3-10]. It is still not clear to what degree the current HSMR and SMR outcomes are attributable to quality of care and can be used to make a meaningful comparison between hospitals. Bottle et al claim however that variations in adjustment methods have a limited impact on the hospitals position relative to funnel plot control limits [11].

One type of potential distortion in establishing comparable HSMRs is caused by the failure to adjust for variations in readmission frequencies. Van den Bosch, et al [10] have demonstrated that the Dutch 2010 HSMR model favoured hospitals with relatively many frequently readmitted patients, regardless of disease, compared to hospitals with many patients who are not frequently readmitted. They were, however, not able to quantify the effect of this upon the HSMR, because the study was restricted to only six hospitals. In a new study more data was made available enabling us to quantify the possible effect in the Netherlands. We were able to compare the HSMR outcomes of the current HSMR model to an alternative model with an additional adjustment for readmission frequency. In essence we asked: Does the new model represent an improvement in the current model, based on c-statistics, goodness of fit and explanatory power? And if so, to what extent do HSMR and SMR outcomes differ, comparing model 1 to model 2?

## Methods

### Setting

For the purpose of this model analysis the DHD group gave its formal permission to use an anonymised LMR dataset from 89 Dutch hospitals covering the years 2005 to 2009. Nineteen hospitals were excluded from the calculation because the quality of their patient registration data was insufficient.

This study excluded any experimental research, either on human or animal subjects.

### The HSMR model used in this study

We performed HSMR calculations using two models, shown as model 1 and model 2.

For *model 1*, we used the Dr Foster model [1], applied in the Netherlands in 2010. This Dutch model included clinical admissions only, so no day cases. Furthermore only *in-hospital *deaths were counted. There are differences between the Dutch and the UK model. The Dutch model used 50 Clinical Classifications Software (CCS) groups based on an ICD-9 coding, whereas the UK used 56 CCS groups based on ICD-10 coding, of which 42 groups were the same as in the Dutch model. Furthermore, the UK model adjusted for palliative care and for the number of previous emergency admissions within one year. The Dutch model did not adjust for either of these.

### HSMR in brief

The ***HSMR ('hospital standardised mortality ratio') ***is the ratio between the observed number of in-hospital deaths and the predicted number of deaths, determined by comparing the patient casemix with the national average. The outcome is standardised around 100. A value above 100 indicates higher mortality than average, below 100, lower mortality. If the calculation is applied to one of the 50 diagnostic groups, then we speak of the SMR ('standardised mortality ratio').

The Dutch HSMR model of 2010 adjusts for the following **casemix properties**: year of discharge, sex, age at admission, admission type, comorbidity (charlsonindex), social deprivation, month of admission, source of referral, diagnostic group, and, in part, for the casemix on the primary diagnostic level.

Each **diagnostic group **is composed of a number of underlying diseases: ICD-9 codes (International Classification of Diseases Ninth edition) as determined by the Clinical Classification Software (CCS). This tool clusters various ICD-9 diagnoses into a manageable number of meaningful categories [12], indicated as 'CCS diagnostic groups'. The selected CCS diagnostic groups have a relatively high mortality and together cover over 80% of the total number of hospital deaths.

For *model 2*, we took model 1 and added an adjustment for the frequency of readmission, as described by Van den Bosch WF, et al [10]. The authors demonstrated that frequently admitting patients was associated with lower mortality ratios *per admission*, for which an additional correction would be needed. In their publication the patients were grouped into 'patient view' classes P(m). We have also applied this to our study. Here the admission frequency m was equal to the number of times a patient was admitted to the same hospital during the five-year study period.

For example, a patient who was admitted ten times during the five-year period to hospital X, to be treated for one or more diseases, possibly with different diagnoses, was part of the patient view class P(10) and not part of any other patient view class. This patient contributed ten admissions to P(10) which were all accounted for in the regression calculation.

We were able to retrieve the number of times that a patient was admitted through the unique patient identification number that all of the 70 hospitals included were using. We have grouped the patient view classes into eight categories: m = 1, m = 2, m = 3, m = 4, m = 5-6, m = 7-9, m = 10-20 and m > 20. This is in order to limit the number of categories for the regression calculation and to avoid categories becoming too small.

### Agreement, or otherwise of the two models

We have calculated to what extent both models did agree, or did not agree, as follows:

1. *Relative change*. To what extent did SMR_model 1 _differ from SMR_model 2 _per CCS diagnostic group, per hospital? Using the formula SMR_delta _= (SMR_model2_/SMR_model1 _-1)*100%, we calculated the shifts for all of the 3500 SMRs (50 diagnostic groups times, 70 hospitals), occurring when changing from model 1 to model 2. We represented these in a frequency distribution. In a similar way we have calculated and represented the 70 shifts that occurred for the HSMRs of the 70 hospitals. We also compared all pairs of death predictions of model 1 and model 2 per admission by calculating regressions coefficients (R^2^) on the SMR level and the HSMR level. These metrics were used as a measure of statistical 'distance' between both models.

2. *Significance scores *of SMRs and HSMRs. We determined per SMR how many hospitals with a significantly high SMR score according to model 1 turned out not to be significantly high according to model 2. And vice versa, in other words: how often a significantly high SMR score in model 2 turned out not to be a significantly high score in model 1. In a similar way we calculated these differences on the hospital level for HSMRs.

### Quality metrics of the two models

We have calculated and compared the quality metrics of the models with respect to:

1. *Discrimination*, expressed in 'c-statistics' on the SMR level and on the HSMR level. This statistical measure indicates how well a regression model is able to predict mortality. Each predicted outcome per admission is compared to the observed outcome: died or survived. A c-statistic of 0.5 has no predictive value: 50% right, 50% wrong. Values above 0.75 suggest good discrimination. A value of 1 is perfect. The overall c-statistic for the Dutch HSMR (model 1) scored above 0.85.

2. *Calibration*, according to Hosmer and Lemeshow. This represents a statistical test for goodness-of-fit that is frequently used in risk prediction models. The test assesses whether or not the observed event rates match expected event rates in subgroups of the model population. The test specifically identifies subgroups as the deciles of fitted risk values. Models in which expected and observed event rates in subgroups are similar, are called well-calibrated [13].

3. *Explanatory power*, using the pseudo R^2 ^statistic according to the 'Nagelkerke R square', in order to assess the degree to which the additional adjustment for readmission changes the unexplained variance in the data.

### Sensitivity of the HSMR model to adjustment for readmission

We have investigated to what extent the model was sensitive to variations in the length of the period under review. We took three scenarios: a period of review of one year (2009), two years (2008-2009) and five years (2005-2009). We then examined the statistical distance between model 1 and model 2, and the three model quality metrics on the HSMR level.

## Results

We analysed the data from 89 hospitals of which 19 were excluded because they had an incomplete registration of the patient identification numbers. Therefore, we included 70 hospitals for the regression calculation, a total of 2494613 admissions.

### Agreement between the two models

**Figure 1:**
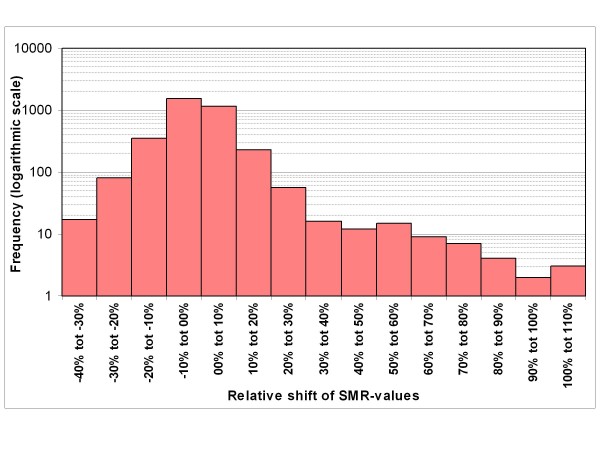
**Frequency distribution of relative changes of all 3500 SMRs when changing from model 1 (Dr Foster model) to model 2 (model with additional adjustment for readmission frequency)**. * Standard deviation amounts to 12.2%.

**Figure 2:**
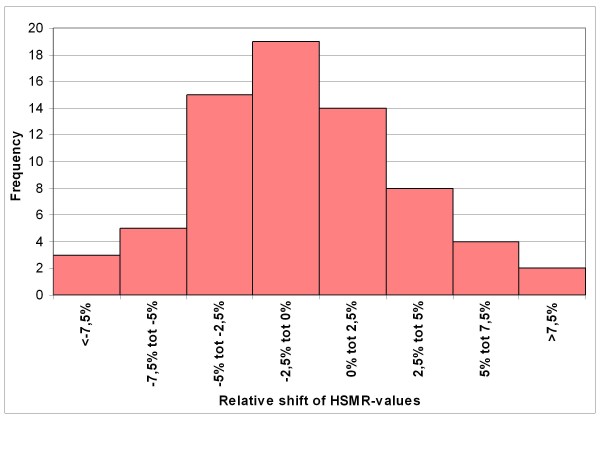
**Frequency distribution of relative changes of the 70 HSMRs when changing from model 1 (Dr Foster model) to model 2 (model with additional adjustment for readmission frequency)**. * Standard deviation amounts to 4.1%.

Figure 1 shows the frequency distribution of the relative changes of the 3500 SMRs, in going from model 1 to model 2. Similarly the changes of the 70 HSMRs of the hospitals are shown in Figure 2. The SMR changes ranged from -39% to +110% and the HSMR changes ranged from -12% to +11%. The standard deviation of the frequency distribution of the changes amounted to 12.2% (roughly 12 SMR points) for the SMRs and to 4.1% (roughly 4 HSMR points) of the HSMRs.

Table 1 shows the agreement that we found between model 1 and model 2, expressed as a statistical distance per SMR. The R^2 ^coefficients varied from 0.60 (COPD) to 0.95 (intracranial injury). For the entire model the coefficient amounted to 0.86.

**Table 1:** A comparison of SMR outcomes of model 1 and model 2

CCS code	CCS-diagnostic group title	Number of admissions	Distance between model 1 and 2 (R^2^)	Number of hospitals with SMR significantly high?		c-statistic for	
				
				Model 1: no model 2: yes	Model 1: yes model 2: no	Model 1	Model 2
2	Septicemia (except in labour)	14647	0.83	2	1	0.760	0.783
12	Cancer of oesophagus	9703	0.74	1	3	0.730	0.763
13	Cancer of stomach	13945	0.75	0	2	0.733	0.779
14	Cancer of colon	45116	0.77	2	5	0.773	0.814
15	Cancer of rectum and anus	22246	0.72	1	3	0.771	0.817
17	Cancer of pancreas	9950	0.76	1	0	0.685	0.704
19	Cancer of bronchus, lung	76714	0.75	4	6	0.792	0.822
24	Cancer of breast	64126	0.74	2	1	0.916	0.850
29	Prostate cancer	24092	0.76	3	1	0.886	0.865
32	Cancer of bladder	44288	0.71	1	0	0.872	0.874
38	Non-Hodgkin's lymphoma	19413	0.69	0	5	0.802	0.841
39	Leukaemias	12466	0.77	0	1	0.781	0.820
42	Secondary malignancies	69303	0.77	5	4	0.748	0.770
44	Neoplasms unspec nature or uncertain behaviour	18982	0.63	2	1	0.805	0.840
50	Diabetes mellitus with complications	36837	0.74	1	0	0.824	0.846
55	Fluid and electrolyte disorders	29705	0.81	0	1	0.795	0.824
59	Deficiency and other anaemia	48896	0.61	0	1	0.756	0.802
85	Coma, stupor and brain damage	4286	0.92	0	0	0.801	0.815
96	Heart valve disorders	30708	0.77	1	2	0.775	0.788
100	Acute myocardial infarction	91802	0.72	1	1	0.740	0.781
101	Coronary atherosclerosis and other heart disease	250183	0.66	1	2	0.784	0.805
103	Pulmonary heart disease	26937	0.78	0	1	0.754	0.776
106	Cardiac dysrhythmias	174923	0.72	1	2	0.828	0.860
107	Cardiac arrest and ventricular fibrillation	8118	0.89	2	2	0.739	0.764
108	Congestive heart failure, non-hypertensive	108541	0.63	4	1	0.637	0.702
109	Acute cerebrovascular disease	102295	0.86	3	0	0.744	0.761
114	Peripheral and visceral atherosclerosis	44090	0.94	1	3	0.908	0.914
115	Aortic, peripheral and visceral artery aneurysms	26538	0.93	1	1	0.871	0.887
116	Aortic & peripheral arterial embolism or thrombosis	32881	0.75	1	1	0.882	0.892
117	Other circulatory disease	20452	0.80	0	1	0.849	0.862
122	Pneumonia	128867	0.74	2	0	0.753	0.785
127	COPD and bronchiectasis	82898	0.60	0	2	0.701	0.761
129	Aspiration pneumonitis	4485	0.79	1	1	0.644	0.688
130	Pleurisy, pneumothorax, pulmonary collapse	24798	0.78	0	0	0.812	0.827
133	Other lower respiratory disease	99460	0.77	1	0	0.854	0.866
145	Intestinal obstruction without hernia	34802	0.76	1	1	0.813	0.836
146	Diverticulosis and diverticulitis	35917	0.70	0	0	0.814	0.828
149	Biliary tract disease	135587	0.80	1	0	0.894	0.899
150	Liver disease, alcohol-related	5133	0.69	0	2	0.678	0.758
151	Other liver diseases	14584	0.80	1	0	0.771	0.807
153	Gastrointestinal haemorrhage	37229	0.71	0	1	0.738	0.764
155	Other gastrointestinal disorders	45487	0.91	0	0	0.907	0.908
157	Acute and unspecified renal failure	8804	0.73	0	0	0.723	0.760
158	Chronic renal failure	14570	0.75	0	2	0.846	0.872
159	Urinary tract infections	62382	0.77	0	0	0.817	0.836
226	Fracture of neck of femur (hip)	74557	0.73	0	2	0.719	0.732
233	Intracranial injury	56535	0.95	0	1	0.939	0.941
237	Complication of device, implant or graft	76079	0.69	0	0	0.821	0.833
238	Complications surgical procedures or medical care	67106	0.76	0	0	0.827	0.833
249	Shock	3150	0.80	1	0	0.716	0.733

	Total all CCS diagnostic groups	**2494613**	**0,86**	**49**	**64**	**0.852**	**0.867**

* Per CCS diagnostic group: number of admissions; statistical distance between model 1 and model 2; number of hospitals with significance differences of SMRs comparing model 1 to model 2, c-statistics for both models. The table includes results of 70 Dutch hospitals over the period 2005 - 2009

Finally, we calculated per SMR for how many hospitals a significantly high SMR score changed to not significantly high from model 1 to model 2 and vice versa; see Table 1. Model 1 indicated 328 SMRs in total as "higher than expected" of which 64 were not indicated by model 2 (20%). On the other hand, model 2 indicated 313 SMRs in total as "higher than expected" of which 49 were not indicated by model 1 (16%). Across hospitals, model 1 indicated that 23 hospitals recorded a higher than expected HSMR of which four were not indicated by model 2 (17%). And, alternatively, model 2 indicated that 23 hospitals were having a higher than expected HSMR of which four were not indicated by model 1 (17%).

### H/SMR model quality metrics

For both models the 'c-statistic' per CCS diagnostic group was calculated, see Table 1. Model 2 scored a higher c-statistic compared to model 1 in 47 groups out of 50. For eighteen of these groups the scores of model 2 exceeded the scores of model 1 by between 0.03 and 0.08. Model 1 had one diagnostic group for which the c-statistic was at least 0.03 better compared to model 2. The c-statistic of the HMSR across all hospitals amounted to 0.852 for model 1 and to 0.867 for model 2 (favourable).

The results of the goodness-of-fit test are shown in Table 2, expressed in the quality metrics of the Hosmer and Lemeshow test. It shows that model 2 was better calibrated compared to model 1: 7434 mismatches (5.75% of the total deaths) for model 1 compared to 5474 (4.23%) mismatches for model 2.

**Table 2:** Contingency table with Hosmer and Lemeshow test for goodness-of-fit

	Died model 1		Died model 2	
	
Decile	Observed	Expected	Observed	Expected
1	138	268	134	231
2	498	786	469	677
3	1057	1464	997	1295
4	1888	2485	1721	2197
5	3609	4038	3002	3544
6	6181	6399	5299	5599
7	10486	10026	8999	8843
8	16797	15578	14890	14337
9	28064	26026	27112	25084
10	60625	62273	66720	67535

Percentage mismatch		5,75%		4,23%

Finally the overall explanatory power expressed in pseudo R^2 ^statistics were calculated. These amounted to 0.259 for model 1 versus 0.282 for model 2 and so favourable to model 2.

### Sensitivity of the model to the review period of adjustment

The overall agreement between models 1 and 2 varied, depending on the review period, from 0.92 (one year) to 0.89 (two years) to 0.86 (five years). So a longer review period corresponded with an increasing divergence between the two models.

Table 3 shows how the overall model quality metrics varied for the three review periods. A longer review period corresponded to an increase of model quality metric performance.

**Table 3:** Comparison of HSMR model quality metrics of model 1 to model 2 for various lengths of review period

	Model 1	Model 2	Difference	In favour of
Overall c-statistic				
1 year period	0.857	0.866	0.009	model 2
2 year period	0.856	0.865	0.009	model 2
5 year period	0.852	0.867	0.015	model 2

**% decile match hosmer lemeshow test**				

1 y period	93.85%	94.13%	0.28%	model 2
2 year period	93.82%	94.41%	0.59%	model 2
5 year period	94.25%	95.77%	1.52%	model 2

**Nagelkerke R square**				

1 year period	0.269	0.285	0.016	model 2
2 year period	0.266	0.284	0.018	model 2
5 year period	0.259	0.282	0.023	model 2

## Discussion

### How should we interpret the differences in model outcomes?

In this study we have calculated and compared the H/SMR outcomes of two models. The disagreement between the two models was substantial, according to the relative changes in HSMRs and SMRs.

The standard deviation of the frequency distribution of HSMR-change amounted to 4 HSMR points, which was substantial compared to the standard deviation of the HSMR-frequency distribution amounting to 14 HSMR points. Moreover, these shifts introduced an uncertainty into the HSMR outcomes that exceded the uncertainty due to chance as indicated by the 95% confidence intervals (CIs): The 95% CI of a HSMR equalled [-2σ, 2σ]. On average for the 70 hospitals over five years, the standard deviation σ of the CI distribution equalled 2.5 HSMR points.

With respect to the SMRs: the statistical 'distance' between SMRs of model 1 and model 2 expressed in R^2 ^coefficients, varied from 0.60 to 0.95. Low scores indicated susceptibility to adjustment for readmissions. On average chronic diseases scored lower compared to acute diseases as to be expected since chronic diseases in particular often result in readmissions. There was no particular peak among any of the main diagnostic groups. The major diagnostic groups like cancer and heart and lung diseases all showed substantial disagreement between model 1 and model 2 due to adjustment for readmission. The results also showed disagreement between the models in designating hospitals as having higher than expected H/SMRs. The percentages of disagreement varied from 16% to 20%.

Bottom-line: all differences in H/SMR outcomes that we found between the two models, cannot be attributed to differences in the quality of care neither to 'chance'. They have to be attributed to the difference in the type of model, because this is the only distinction applied in producing the results from the same set of admissions. This implies that publicised good and bad practices, linked to hospital rankings based on HSMRs, can differ substantially depending on the choice of model.

### How do readmissions affect H/SMRs and how do they occur?

We asked: how did readmissions impact the H/SMR, how did readmissions occur and why did hospitals differ in this respect?

For example: endlessly readmitting the same patient in the same hospital continuously increased the denominator (predicted death) of the calculated HSMR for that hospital. We have observed numerous cases where the contribution to the denominator on behalf of a single patient over the years aggregated to numerical values of 3 to 4. However a patient could only die once and so any patient could maximally contribute a numerical value of 1 to the numerator. Consequently this effect lowered the HSMR ratio and favoured hospitals which had many frequently readmitted patients.

There are many types of mechanisms that explain the variation in the frequency of clinical readmission. These could have been administrative in nature, for example differences in administrating chemotherapy [10]. There were also many systematic differences in treatment practice. For example one hospital applied and recorded a different number of clinical admissions than another hospital did for the same combination of diagnosis and treatment applied to the same patient. Differences also occurred as a consequence of transferring patients back and forth. Poor care during the initial admission may also have triggered readmissions later. As a consequence all these examples of increased readmissions were being 'rewarded' by model 1 while model 2 was correcting for this phenomenon.

The analysis showed that the combined effect of all these mechanisms was not restricted to some of the diagnostic groups, but had an impact upon most of them. Clearly the HSMR model was susceptible to an adjustment for the frequency of readmissions in the way we have defined it.

The analysis also showed that reducing the review period from five to two years and from two to one year, resulted in a substantial reduction of this susceptibility. This phenomenon may be explained as follows: It may take several years before the readmissions of a patient constitute a substantial sequence of admissions. Furthermore a sequence might also have started long before the analysis period started. In these cases apparently one year was not a sufficiently long time period to capture sequences with a substantial amount of admissions. By looking at various years these sequences became better visible.

### Choice of model: consequences for usage of H/SMR as indicator

We considered which of the two models would be more favourable to use and why. Since the model characteristics with regard to discrimination, calibration and explanatory power all were in favour of model 2, we recommend using model 2, although this preference does not necessarily invalidate model 1. HSMR models in other countries may need a similar additional adjustment. The impact of readmissions on the current HSMR in the UK is not clear. The UK model adjusts for readmissions for emergency cases. However for non-emergency cases the model does not adjust for readmissions. Moreover, admissions with different primary diagnoses are not counted as readmissions as well. Furthermore the UK model is restricted to a maximum review period of one year, which is too short for the readmission effect to become visible as demonstrated in our study.

A more fundamental issue than the choice of model however, is the fact that two comparable models, such as model 1 and model 2, may deliver such divergent outcomes. Since there is no 'gold standard' one cannot state that the one is false and the other one is true. This finding reveals an uncertainty in HSMR outcomes that exceeds the amount of uncertainty introduced by chance expressed in 95% confidence intervals over the five-year period.

HSMRs and SMRs are increasingly used by physicians to improve the quality of care in their hospitals. In particular diagnostic groups with higher than expected mortality are the subjects of investigation. However, in a lot of cases, the designated significance of these groups may depend on the regression model used, not only on the confidence intervals. The same is applicable for the current HSMR scores in the Netherlands. Physicians and hospital managers should be aware of this phenomenon.

### Limitations of the study

A limitation of this study was the fact that we had to exclude 19 hospitals and so the HMSR calculation was based on roughly 80% of the Dutch hospitals. Nevertheless, we think the results of our study were representative of the Netherlands and have clearly demonstrated the impact upon the HSMR of not adjusting for frequency of readmissions.

Another limitation was the fact that the Dutch HSMR only accounts for in-hospital mortality and not for 30-day mortality, which may favour hospitals with shorter lengths of stay [14]. However since the effects, demonstrated in our study, particularly concerned the phenomenon of the frequently returning patient, and so no 30-days deaths, we think this potential bias does not significantly change our conclusions.

## Conclusions

The HSMR model for the Netherlands was sensitive to adjustment for frequency of readmissions. Model 1, without adjustment, compared to model 2, with adjustment, produced substantially different HSMR outcomes. The uncertainty introduced by these differences exceeded the uncertainty indicated by the 95% confidence intervals. Since model 2 turned out to be more accurate compared to model 1 with regard to c-statistics, goodness of fit and explanatory power, the authors would prefer to apply adjustment for frequency of readmission. Parties, also in other countries, making use of HSMR-calculations may decide for themselves whether or not it is clinically relevant in their situation to include frequent admissions in the model. In case of adjustment, a review period of at least three years is advisable.

## Competing interests

The authors declare that they have no competing interests.

## Authors' contributions

All authors have contributed significantly to the conception and design of the study. WB collected and analysed the data and produced the first draft of the manuscript. PS conducted the regression calculations. All authors revised the manuscript critically for important intellectual content and gave final approval of the version to be published.

## Pre-publication history

The pre-publication history for this paper can be accessed here:

http://www.biomedcentral.com/1472-6963/12/91/prepub

## References

[B1] Jarman B Pieter D van der Veen AA The hospital standardised mortality ratio: a powerful tool for Dutch hospitals to assess their quality of care? Qual Saf Health Care 2010 19 9 13 20172876 10.1136/qshc.2009.032953 PMC2921266

[B2] The 2010 Dr Foster Hospital Guide http://www.drfosterhealth.co.uk (accessed 12 May 2011)

[B3] Lilford R Pronovost P Using hospital mortality rates to judge hospital performance: a bad idea that just won't go away BMJ 2010 340 c2016 20406861 10.1136/bmj.c2016

[B4] Black N Assessing the quality of hospitals BMJ 2010 340 c2066 20406863 10.1136/bmj.c2066

[B5] Penfold RB Do hospital standardised mortality ratios measure patient safety? HSMRs in the Winnipeg Regional Health Authority Healthcare Papers 2008 8 4 8 23 18667867 10.12927/hcpap.2008.19972

[B6] Van den Bosch WF Roozendaal KJ Silberbusch J Wagner J Variation in coding patient data impacts hospital standardised mortality ratios (HSMR) Ned Tijdschr Geneeskd 2010 154 A1189 in Dutch 20178667

[B7] Van den Bosch WF Graafmans WC Pieter D Westert GP Cardiac centres and the HSMR. The impact of special medical procedures upon the hospital standardised mortality ratio Ned Tijdschr Geneeskd 2008 in Dutch 1221 7 in Dutch 18578452

[B8] Mohammed MA Deeks JJ Girling A Rudge G Carmalt M Stevens AJ Lilford RJ Evidence of methodological bias in hospital standardised mortality ratios: retrospective database study of English hospitals BMJ 2009 338 b780 19297447 10.1136/bmj.b780 PMC2659855

[B9] Lilford R Mohammed MA Spiegelhalter D Thomson R Use and misuse of process and outcome data in managing performance of acute medical care: avoiding institutional stigma Lancet 2004 363 1147 1154 15064036 10.1016/S0140-6736(04)15901-1

[B10] Van den Bosch WF Kelder HC Wagner C Predicting hospital mortality among frequently readmitted patients: HSMR biased by readmission BMC Health Serv Res 2011 11 57 21401936 10.1186/1472-6963-11-57 PMC3063816

[B11] Bottle A Jarman B Aylin P Hospital standardized mortality ratios: sensitivity analysis on the impact of coding Heal Serv Resdoi:10.1111/j.1475-6773.2011.01295.x 10.1111/j.1475-6773.2011.01295.x PMC3393030 21790587

[B12] Clinical Classifications Software (CCS) for ICD-9-CM Fact Sheet http://www.hcup-us.ahrq.gov/toolssoftware/ccs/ccsfactsheet.jsp#what

[B13] Hosmer DW Lemeshow S Applied Logistic Regression 2000 New York: Wiley ISBN 0471615536

[B14] Drye EE Normand SLT Wang Y Comparison of hospital risk-standardized mortality rates calculated by using in-hospital and 30-day models: an observational study with implications for hospital profiling Ann Intern Med 2012 156 no. 1 Part 1 19 26 22213491 10.1059/0003-4819-156-1-201201030-00004 PMC3319769

